# Fine-scale mapping of *Schistosoma mansoni* infections and infection intensities in sub-districts of Makenene in the Centre region of Cameroon

**DOI:** 10.1371/journal.pntd.0010852

**Published:** 2022-10-13

**Authors:** Estelle Mezajou Mewamba, Arnol Auvaker Zebaze Tiofack, Cyrille Nguemnang Kamdem, Esthelline Yangea Tchounkeu, Rostand Joël Atiokeng Tatang, Loic Edmond Tekeu Mengoue, Mureille Carole Tchami Mbagnia, Flobert Njiokou, Miriam Casacuberta-Partal, Hilaire Macaire Womeni, Gustave Simo, for the TrypanoGEN+ research group of the H3Africa consortium

**Affiliations:** 1 Molecular Parasitology and Entomology Unit, Department of Biochemistry, Faculty of Science, University of Dschang, Dschang, Cameroon; 2 Research Unit of Biology and Applied Ecology, Department of Animal Biology, Faculty of Science, University of Dschang, Dschang, Cameroon; 3 Parasitology and Ecology Laboratory, Department of Animal Biology and Physiology, Faculty of Science, University of Yaoundé I, Yaoundé, Cameroon; 4 Centre for Research in Infectious Diseases, Yaoundé, Cameroon; 5 Department of Parasitology, Leiden University Medical Center, Leiden, The Netherlands; 6 Unité de Recherche de Biochimie, des plantes Médicinales, des Sciences alimentaires et Nutrition, University of Dschang, Dschang, Cameroon; University of California Berkeley School of Public Health, UNITED STATES

## Abstract

**Background:**

Schistosomiasis control relies mainly on mass drug administration of Praziquantel (PZQ) to school aged children (SAC). Although precision mapping has recently guided decision making, the sub-districts and the epidemiological differences existing between bio-ecological settings in which infected children come from were not taken into consideration. This study was designed to fill this gap by using POC-CCA and KK to comparatively determine the prevalence and infection intensities of *Schistosoma mansoni (S*. *mansoni)* and to perform fine-scale mapping of *S*. *mansoni* infections and its infection intensities with the overarching goal of identifying sub-districts presenting high transmission risk where control operations must be boosted to achieve schistosomiasis elimination.

**Methodology:**

During a cross- sectional study conducted in Makenene, 1773 stool and 2253 urine samples were collected from SAC of ten primary schools. *S*. *mansoni* infections were identified using the point of care circulating cathodic antigen (POC-CCA) and Kato-Katz (KK) test respectively on urine and stool samples. Geographical coordinates of houses of infected SAC were recorded using a global position system device. Schistosome infections and infection intensities were map using QGIS software.

**Results:**

The prevalence of *S*. *mansoni* inferred from POC-CCA and KK were 51.3% and 7.3% respectively. Most infected SAC and those bearing heavy infections intensities were clustered in sub-districts of Baloua, Mock-sud and Carrière. Houses with heavily-infected SAC were close to risky biotopes.

**Conclusion:**

This study confirms the low sensitivity of KK test compared to POC-CCA to accurately identify children with schistosome infection and bearing different schistosome burden. Fine-scale mapping of schistosome infections and infection intensities enabled to identify high transmission sub-districts where control measures must be boosted to reach schistosomiasis elimination.

## Author summary

Although some disparities in terms of prevalence and infection intensities have been acknowledged within and between schistosomiasis endemic areas, the current control measures did not take into consideration the disparities within endemic areas. To improve the control of schistosomiasis, a fine-scale mapping of schistosome infections and their intensities were undertaken using KK in comparison to POC-CCA to identify sub-districts with potential high transmission risk and where control operations must be boosted to achieve elimination. After the identification of schistosome infections by the point of care circulating cathodic antigen (POC-CCA) and Kato-Katz (KK) test, the geographical coordinates of each infected child’s house were recorded using a global position system device (GPS). QGIS software was used to create a map showing schistosome infections and their infection intensities. Our results showed that the majority of infected children and those bearing heavy infection intensities were clustering mostly in Baloua, Carrière and Mock-Sud sub-districts of Makenene while children with light and moderate infection intensity were widely distributed and far away from risky biotopes. The fine-scale mapping of schistosome infections and their infection intensities enable to identify hotspot transmission sites where control strategy must be boosted to achieve the elimination of intestinal schistosomiasis in Makenene.

## Introduction

Schistosomiasis remains a major public health problem in people living in vulnerable communities and where sanitation and water supply are insufficient [1–3]. About 700 million people are at risk of schistosome infections and more than 200 million are currently infected [4]. In addition, more than 90% of the global schistosomiasis burden occurs in Africa alone with about 200 000 deaths per year [5,6].

Several strategies including preventive chemotherapy (PC), water sanitation and hygiene, snail control and the dissemination of information, education, and communication have been developed to fight against schistosomiasis [7,8]. However, over years, the control of schistosomiasis continues to rely mainly on PC in which school-aged children are massively treated with Praziquantel (PZQ). Although this strategy enabled to reduce the disease prevalence as well as the infection intensity in most affected communities [9,10], updating epidemiological data on schistosomiasis remains important for evaluating and monitoring the success of control strategies. Mapping accurately the disease prevalence and infection intensities in affected communities could help to determine the number of treatment rounds and to monitor the progress towards schistosomiasis elimination [9,11–13]. Although mapping of schistosome infections has been undertaken in most endemic areas, the bio-ecological differences that exist between sub-districts were not often taken into consideration.

Reaching the control targets set into WHO Road Map and ending the neglect in order to attain the Sustainable Development Goal of good health and well-being requires sustainable framework for action against neglected tropical diseases that was developed for 2021 to 2030 [3]. It is in this framework that fine mapping of schistosome infections and their infection intensities appears crucial for the identification of sub-districts showing potentially high schistosomiasis transmission sites. Recently, the use of precision mapping enabled to deeply understand the distribution of schistosomiasis and also to guide decision making in boosting the elimination of schistosomiasis in some endemic areas of Cameroon [14]. In previous studies, the sub-districts from whom the infected children come from were not taken into consideration because all data were collected in schools. Considering the sub-district of each infected child appears fundamental for fine-scale mapping of schistosome infections. Moreover, in low endemicity areas and especially those subjected to several years of mass administration of PZQ and where the overall schistosomiasis prevalence has been considerably reduced like in the endemic area of Makenene in Cameroon, the reported low sensitivity of KK does not play in favor of its use for fine-scale mapping of schistosomiasis. It is in this light that the point of care circulating cathodic antigen (POC-CCA) and Kato-Katz (KK) test were used to comparatively determine the prevalence and infection intensities of *S*. *mansoni* and subsequently perform fine-scale mapping of *S*. *mansoni* infections and its infection intensities with the overarching goal of identifying sub-districts presenting high schistosomiasis transmission risk where control operations must be boosted to achieve schistosomiasis elimination.

## Materials and methods

### Ethical statement

The study was approved by the National Ethics Committee for research on human health of the Ministry of Public Health of Cameroon with the reference number N°2020/08/1284/CE/CNERSH/SP. The review board of the Molecular Parasitology and Entomology Sub-unit of the Department of Biochemistry of the Faculty of Science of the University of Dschang gave its approval. Field survey was conducted in schools with the approval of the administrative authorities, school inspectors, directors and teachers. Parents or guardians of participating children approved their participation by signing the informed consent form on their behalf. In addition, children of 10 to 14 years signed an assent form while for younger ones, only the consent form signed by their parents or guardians was considered. After detailed explanation of the objectives, the procedures and the potential risks and benefits, each child was free to choose whether to participate in the study. Data were anonymised during analyses.

Results of parasitological and immunological tests were communicated to parents or guardians and all children found with schistosome infections were treated with PZQ (40mg/Kg body weight) following WHO recommendations [11].

### Study area

**Fig 1:**
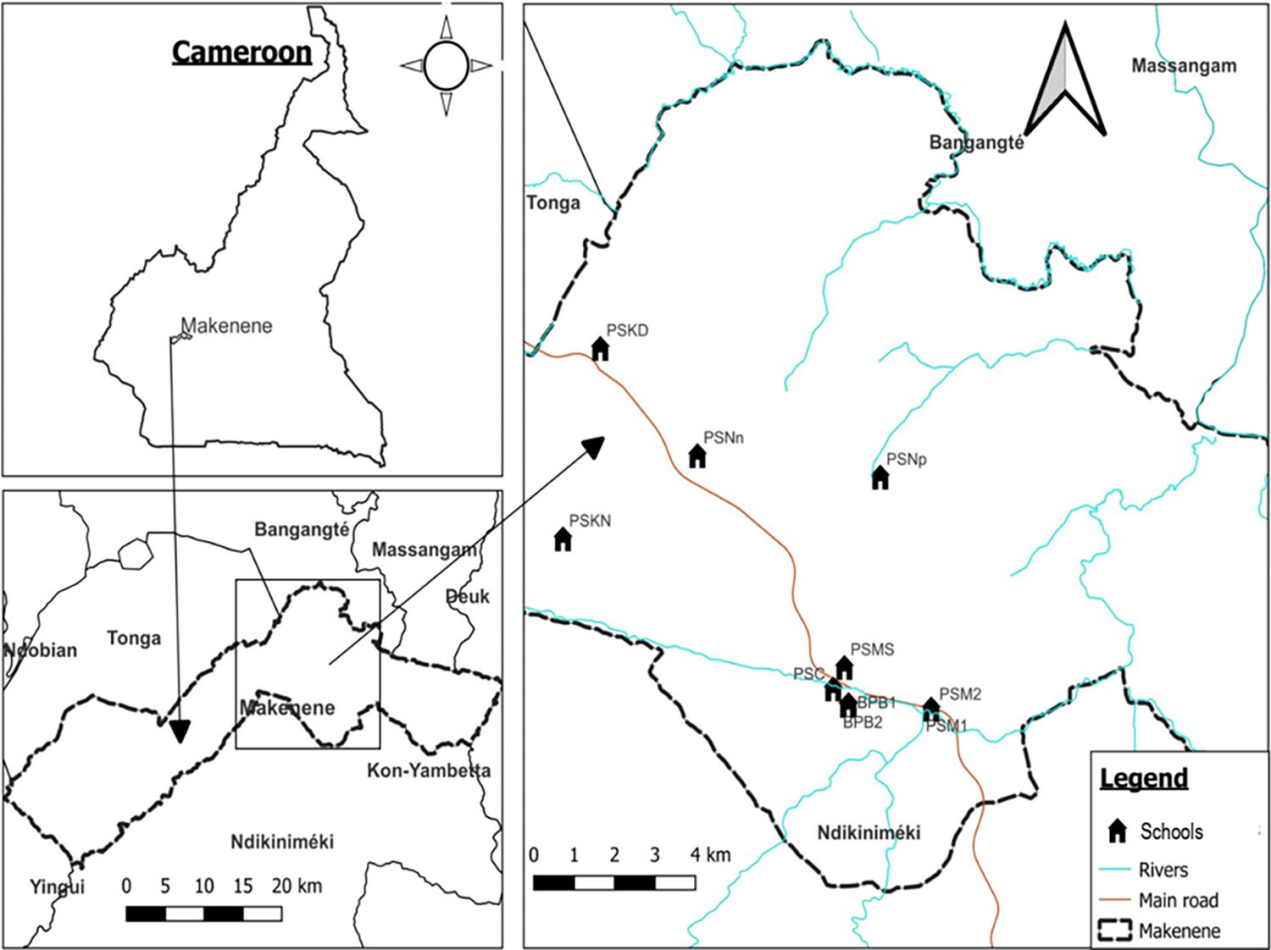
Map of Makenene showing schools where children were sampled (map obtained from: https://qgis.org/downloads/QGIS-OSGeo4W-3.16.10-1-Setup-x86_64.exe with layers downloaded using the link: https://www.diva-gis.org/gdata; consulted, 14/09/2021). BPB1: Government bilingual primary school of Baloua group 1; BPB2: Government bilingual primary school of Baloua group 2; PSM1: Public school of Makenene group 1; PSM2: Public school of Makenene group 2; PSNn: Public school of Nyokon; PSKD: Public school of Kinding Nde; PSKN: Public school of Kinding Ndjabi; PSMS: Public school of Mock-Sud; PSC: Public school of Carrière; PSNp: Public school of Ngokop.

This study was carried out in November 2020 in 8 sub-districts of Makenene which is a rural locality of the Mbam and Inoubou Division of the Centre Region of Cameroon (Fig 1). Each sub-district or bio-ecological setting can be seen here as area or type of surrounding where interactions between human with both biotic (presence of snails) and abiotic factors (presence of standing, clear or fresh water) could favour or not the transmission of schistosomiasis. Makenene is located at 200 km in the north-west of Yaoundé and has about 16,000 inhabitants. It belongs to the Ndikinimeki Health District and it is delimited in the north by the council of Massagam, the south by the council of Yabassi and Ndikimimeki, the east by the council of Deuk and Konyambetta and finally in the west by the council of Tonga. It has an equatorial climate with 4 seasons: two dry seasons from November to March and from mid-May to mid-August and two rainy seasons from August to November and March to May. It has a dense hydrographic network containing several rivers including the Mock, Makombé, Makongo, Managa, Mefom, Niep, Bokokeut, Kyakan, Mayi, Molo, Makam, Sinsam, Bambi, Djanka and Noum. Inhabitants practice petty trading and farming and the main crops are cassava, corn, groundnuts, yam, cocoa and palm nuts. *Schistosoma mansoni* infections have been reported in sub-districts of Makenene for more than 30 years [15–17]. In this locality, the control against schistosomiasis relies on annual distribution of PZQ to school-aged children by the national control program for schistosomiasis and soil-transmitted helminthiasis [18]. Although schistosomiasis is still considered as an important public health problem, the overall disease prevalence and infection intensity has been reduced in the endemic area of Makenene.

### Study design and population

This cross-sectional study involved school-aged children attending primary schools of 8 sub-districts of Makenene. These sub-districts have 14 primary schools including 4 privates and 10 public schools. Out of these 14 schools, authorisations to perform this study were obtained for 10 public schools located in 8 sub-districts. In six sub-districts including Kinding Nde, Kinding Ndjabi, Nyokon, Carrière, Ngokop and Mock-sud, only one school was found in each of them. All the six schools were enrolled in this study. However, in the sub-districts of Makenene and Baloua, 4 primary schools (two privates and two publics) were found in each of these two sub-districts due to the high density of population compared to other sub-districts. The two public schools for which authorizations were obtained and that contained the majority of school-aged children of the sub-district were selected for this study. Participants were therefore children attending ten primary schools of Makenene (Fig 1): the government bilingual primary school of Baloua group 1 (BPB1), the government bilingual primary school of Baloua group 2 (BPB2), the public school of Makenene group 1 (PSM1), the public school of Makenene group 2 (PSM2), the public school of Nyokon (PSNn), the public school of Kinding Nde (PSKD), the public school of Kinding Ndjabi (PSKN), the public school of Mock-Sud (PSMS), the public school of Carrière (PSC) and the public school of Ngokop (PSNp). The sample size was made up of all school-aged children of 5 to 15 years attending the 10 primary schools and for whom a signed inform consent form was obtained from their parents or guardians.

### Sample collection

Before sampling, the objective of the study was explained to directors and teachers of each school. The sampling day was communicated to children by their teachers. During the sampling day, orientations about the sampling process were given to directors and teachers before sampling. Each child who provided assent and had a signed inform consent from their parents or guardians was invited to provide urine and stool samples in clean and well-labelled plastic containers that were given on the sampling day. Approximately 50 ml of urine and 5 to 7g of stool were collected from children between 9:00 am and 2:00 pm. These samples were immediately transferred to the local health centre where the POC-CCA and KK tests were performed respectively on urine and stool samples. The samples were kept at room temperature and completely processed within 12 hours following their collection.

### Detection of *S*. *mansoni* infection using the Kato-Katz technique

Schistosome eggs were detected using the protocol described by Katz et al. [19]. From each stool sample, a single KK thick smear slide was prepared with 41.7 mg of stool using Sterlitech kit (Lot: XGACAI). Briefly, a small quantity of stool was transferred onto a piece of scrap paper. Thereafter, a nylon mesh was pressed on the top of the faecal sample and a small plastic spatula was used to scrap the sieved material of the nylon screen. Subsequently, the well of the KK template was placed on a clean microscopic slide and completely filled with sieved faecal material. The template was removed without disturbing the calibrated faecal material. Thereafter, the slide was covered with a cellophane strip pre-soaked for 24 hours in glycerol-malachite green solution (a mixture of 100 mL of mineral water, 100 mL of glycerin and 1 mL of 3% green malachite solution). The stool was spread onto a thick smear by inverting the microscope slide and pressing the stool sample against the cellophane on a smooth surface. The slides were observed after 24 hours under the microscope at a magnification of ×10 objective. Eggs of *S*. *mansoni* were morphologically identified by examining each microscope slide.

### Detection of *S*. *mansoni* infection using POC-CCA test

Circulating cathodic antigen was detected using POC-CCA (Rapid Medical Diagnostics, Pretoria, South Africa, batch no 190411032). The POC-CCA test was performed according to the manufacturer instructions. Briefly, two drops of urine sample were put in the well of the POC-CCA cassette. After complete absorption of the urine sample for exactly 20 minutes, the POC-CCA cassette was visualized. A test was considered valid if the control line turned a dark pink colour. If not, the sample was re-tested with a new cassette. Any cassette that was not read at 20 minutes was considered as invalid and therefore, re-tested [20].

### Quantification of CCA on POC-CCA strips

The amount of CCA in each urine sample was determined by reading each POC-CCA cassette with a lateral flow strip reader (ESEQuant LR3 reader) and the visual scoring system as described by Mewamba et al. [21] and Casacuberta et al. [22] respectively.

### Quantification of CCA using visual scoring system

For the visual scoring system, the intensity of bands appearing on POC-CCA cassette were compared to those of G-scores which are series of 10 artificial cassettes containing inkjet-printed strips with different line intensities named G1 to G10 [22]. The test line of each POC-CCA cassette was compared to the intensity of artificial cassettes and then, classified as negative, “trace”, 1+, 2+ or 3+ depending of their correspondence with G-scores [22].

### Quantification of CCA using a lateral flow strip reader

The ESEQuant LR3 reader was designed to quantify target analytes on Lateral Flow Test strips including the CCA strips. Before its use, the reader was calibrated using the S-series provided by the Department of Parasitology of the Leiden University Medical Center of Leiden in the Netherlands. During CCA quantification, each cassette was introduced into the drawer of the ESEQuant LR3 reader and the intensity of CCA on each POC-CCA cassette was estimated and expressed in millivolts as described by Mewamba et al. [21]. Each measurement expressed in millivolts was proportional to the intensity of the band reflecting the level of CCA on the strip or in urine sample [23]. All samples for which the reader provided a signal intensity of the POC-CCA test line greater than 40 millivolts were considered positive or having CCA of *S*. *mansoni*. The POC-CCA data generated from the visual scoring system were used to map the infection intensities of *S*. *mansoni*.

### Collection of geographical coordinates of houses of children with *S*. *mansoni* infections

After performing KK and POC-CCA tests on all stool and urine samples, infected children were identified. Thereafter, each child identified as positive was accompanied home with at least one member of the research team in order to record the geographical coordinates of the house using a global position system (eTrex, Garmin International, Olathe, KS, USA). Recording was performed after detailed explanation of the purpose of the study and the approval of the head of the family. Other environmental factors that could favour snails’ development and the transmission of schistosomiasis such as the presence of river, swimming area, sites to wash clothes and fetch water were recorded by collecting their geographical coordinates using a global position system.

### Mapping the prevalence and infection intensities of *S*. *mansoni*

Cameroon map containing the administrative divisions was downloaded using the link https://www.diva-gis.org/gdata (consulted, 14/09/2021). Thereafter, the geographical information layers on the road network and the hydrographic network were projected onto the map using the geographical information system software (QGIS v.3.16.10 ’Hannover’; QGIS Development Team, https://qgis.org/downloads/QGIS-OSGeo4W-3.16.10-1-Setup-x86_64.exe). In addition to that, the geographical coordinates of schools, houses of infected children and those of risky biotopes (areas where snails can be found such as standing water, clear water and freshwater and where children perform risky activities like swimming, bathing, fetching water, washing clothes or dishes) for schistosomiasis transmission were inserted onto the map of endemic regions using the same QGIS v.3.16.10 software. This software was subsequently used to map *S*. *mansoni* infections and its infection intensities inferred from KK and POC-CCA tests.

### Statistical analysis

Data were computed using SPSS 22.0 (SPSS Inc., Chicago, Illinois, USA) and R software (R3.6.1 "Action of the Toes"). The chi-square test of equality of proportion and Fisher exact test were used to compare the prevalence of *S*. *mansoni* infections between age groups, schools and sexes. Children were stratified into two age groups (5–10 and 11–15 years). The mean infection intensities (expressed in millivolts for POC-CCA and eggs per gram of faeces for KK) between sexes or age groups were compared using the Student’s *t*-test. The Analysis of variance test (ANOVA) was used to compare the arithmetic mean of infection intensities (expressed in millivolt for POC-CCA or eggs per gram of faeces for KK) according to schools. The infection intensities were reported as mean of CCA or EPG ± standard deviation (SD). Statistical tests were evaluated at 95% confidence interval (CI). The significance was set at P < 0.05. The 95% confidence interval obtained in this study was calculated using the MedCalc online software (https://www.medcalc.org/calc/rate_ci.php). This 95% CI is a range of values for which the true means of either prevalence or infection intensity are reliable with 95% of confidence. The intensity of *S*. *mansoni* infection was expressed in eggs per gram of faeces (EPG) and then, classified according to WHO guidelines as light, moderate or heavy infections when the number of eggs per gram of stool was respectively <100 EPG, 100–400 EPG and >400 EPG [11].

The sensitivity, specificity and accuracy of POC-CCA test were assessed as described by Baratloo et al. [24] using KK as “gold standard test”. The strength of agreement between POC-CCA and KK was assessed using Kappa statistics. The value of Kappa was interpreted according to the classification of Landis and Koch [25]. The test was considered significant for a P value below 0.05. The relationship between infection intensities inferred from the reader (expressed in millivolts) and KK (expressed in EPG) was estimated using the Spearman correlation test.

The global clustering or spatial correlation between houses of infected children harbouring different infection intensities was assessed by determining the Moran’s I correlation coefficient as described by Cliff and Ord [26]. In addition to that, the local Moran’s Ii correlation coefficient proposed by Anselin [27] was used to determine local clustering of houses of children harbouring different infection intensities of *S*. *mansoni*. Maps showing global and local clustering were created using ArcGIS software version 10.8 (ESRI Inc. Redlands, CA, USA.)

The global moran’s correlation coefficient I is a measure of spatial auto-correlation. It estimates the general strength of spatial auto-correlation among sub-districts while its local equivalent estimates the spatial auto-correlation between houses of a sub-district and that of neighboring sub-districts. Statistical values from the assessment of Moran’s I coefficient were interpreted as described by Faisal and Ghaleb [28]. The values of Moran’s correlation coefficient vary from -1 to +1. When I is higher than zero with a significant *P value*, houses of children having the same infection intensities seem to clustering together. For a value of I lower than zero with a significant *P value*, houses of infected children seem to clustering together without clustering of houses of children having the same level of infection intensities. If the value of I is zero, there is no correlation.

The distances between houses of infected children or schools and the nearest river or water points were determined using the QGIS software (QGIS v.3.16.10 ’Hannover’; QGIS Development Team). Linear regression model was used to see if the distances of schools to the nearest water points were associated with the infection intensities of *S*. *mansoni*. The ANOVA test was used to compare the mean distance between the nearest water points and houses of infected children carrying different infection intensities.

## Results

### Characteristics of the study population

For this study, 2317 children from ten schools provided urine and/or stool samples. The percentage of children who declined to participate or for whom the parents or guardians did not provide a signed inform consent was below 0.8%. Out of these 2317 children, 2253 (97.2%) provided urine and 1773 (76.5%) stool samples. The number of children who provided both urine and stool samples was 1709 (73.7%). Five hundred and forty four (23.4%) children provided only urine samples while 64 (2.7%) children provided only stool samples. The study population consisted of 1220 (52.6%) males and 1097 (47.3%) females. The mean age of children examined was 8.5 ± 2.2 (95% CI 8.4–8.6) years: 8.6 ± 2.3 (95% CI 8.6–9.1) for boys and 8.3 ± 2.1 (95% CI 7.9–8.5) for girls. Boys were significantly older than girls (*t = 3*.*5*, *P < 0*.*001*). Although children from 5 to 15 years old were recruited for this study, the percentage of participating children vary significantly (***χ2*** *= 1179*.*9*, *P < 0*.*0001*,) according to ages (Table 1).

**Table 1:** Prevalence of S. *mansoni* infections according to age.

Age	NCE (%)	NUA	POC-CCA+ (%)	NSA	KK+ (%)
5	111 (4.8)	105	55 (52.3)	84	4 (4.7)
6	463 (20)	442	203 (45.9)	347	16 (4.6)
7	299 (12.9)	287	133 (46.3)	225	9 (4)
8	349 (15.1)	342	163 (47.6)	264	17 (6.4)
9	302 (13)	298	146 (48.9)	233	14 (6)
10	339 (14.6)	335	185 (55.2)	273	30 (10.9)
11	210 (9.1)	206	124 (60.2)	161	16 (9.9)
12	147 (6.3)	143	91 (63.6)	114	11 (9.6)
13	59 (2.5)	57	32 (56.1)	46	5 (10.8)
14	24 (1)	24	18 (75)	17	5 (29.4)
15	14 (0.6)	14	7 (50)	9	2 (22.2)
Total	2317	2253	1157	129	129
P-value	< 0.0001		0.0002		0.0002
χ2	1179.9		33.6		33.2

NCE: Number of children enrolled: NUA: Number of urine samples analysed; NSA: Number of stool samples analysed; POC-CCA+: Number of children positive for the POC-CCA test; KK+: Number of children positive for the Kato Katz

### Prevalence and infection intensity of *Schistosoma mansoni* inferred from Kato Katz

#### Prevalence of Schistosoma mansoni inferred from Kato Katz

From 1773 children whose stool samples were examined by KK, 129 harboured *S*. *mansoni* eggs; giving thus a prevalence of 7.3% (95% CI 6.1–8.6). The highest prevalence of 24.1% was recorder in the public school of Carrière (95% CI: 18.6–30.6) while no *S*. *mansoni* egg was observed in stool samples of children attending the public schools of Nyokon, Kinding Nde, Kinding Ndjabi and Mock-sud (Table 2). Comparing the prevalence of *S*. *mansoni* infections inferred by KK, significant difference (*χ*^*2*^ *= 165*.*7*, *p < 0*.*0001*, *df = 9*) was observed between schools (Table 2).

**Table 2:** Prevalence and infection intensity of *S*. *mansoni* infections according to schools.

School	NUA	POC-CCA test				NSA	KK tests			
		**POC-CCA+ (%)**	**95% CI** ^**a**^	**Mean CCA value (mv) ± SD**	**95% CI** ^**b**^		**KK+ (%)**	**95% CI** ^**a**^	**Mean value of EPG ± SD**	**95% CI** ^**b**^
**BPB1**	243	139 (57.2)	48.0–67.5	228.3±183	225.8–230.8	193	19 (9.8)	5.9–15.3	256.4±289.7	249.2–263.7
**BPB2**	282	168 (59.5)	50.9–69.3	239.1±164	236.8–241.4	227	20 (8.8)	5.3–13.6	475.20±782.221	465.7–484.8
**PSM1**	257	112 (43.5)	35.8–52.4	116±94.2	114–118	139	8 (5.7)	2.49–11.3	108±142.8	100.9–115.4
**PSM2**	196	94 (47.9)	38.7–58.7	125±11.4	122.8–127.3	155	7 (4.5)	1.82–9.3	44.5±35.1	39.7–49.8
**PSNn**	218	81 (37.1)	29.5–46.1	82.5±44.9	80.5–84.5	174	0 (0)*	0.0	0.0	0.0
**PSKD**	93	32 (34.4)	23.5–48.5	82.6±31.6	79.4–85.8	80	0 (0)*	0.0	0.0	0.0
**PSKN**	150	54 (36)	27.0–46.9	80.4±46.5	78–82.8	141	0 (0)*	0.0	0.0	0.0
**PSMS**	96	54 (56. 2)	42.2–73.4	156.7±142.9	153.4–160.1	77	0 (0)*	0.0	0.0	0.0
**PSC**	376	273 (72. 6)	64.2–81.7	257.4±154.5	255.5–259.3	274	66 (24.1)	18.6–30.6	617.8±1150.9	611.8–623.8
**PSNp**	342	150 (43.8)	37.1–51.4	164.2±143.5	162.2–166.3	313	9 (2.8)	1.3–5.4	50.6±34.8	46.1–55.5
**Total**	2253	1157 (51.3)	48.4–54.4	184.7±153.2	183.9–185.5	1773	129(7.3)	6.0–8.6	440.2±907.8	436.5–443.8
**P-value**		< 0.0001^a^		< 0.0001^b^			< 0.0001^a^		0.19^b^	
**χ** ^**2**^		137.1, df = 9		-			165.7, df = 9		-	
**F**		-		28.3,df = 9			-		1.5, df = 5	

BPB1: Government bilingual primary school of Baloua group 1; BPB2: Government bilingual primary school of Baloua group 2; PSM1: Public school of Makenene group 1; PSM2: Public school of Makenene group 2; PSNn: Public school of Nyokon; PSKD: Public school of Kinding Nde; PSKN: Public school of Kinding Ndjabi; PSMS: Public school of Mock-Sud; PSC: Public school of Carrière; PSNp: Public school of Ngokop; NUA: Number of urine samples analysed; NSA: Number of stool samples analysed; POC-CCA+: Number of children positive for POC-CCA test; KK+: Number of children positive for the Kato Katz; CCA: Circulating cathodic antigen; EPG: egg per gram of stool; SD: Standard deviation, ^a^: P-value for chi-square test; ^b^: P- value for ANOVA test; *:The prevalence of parasite species was 0% and the 95% CI could not be calculated; 95% CI^a^: 95% confidence interval associated to prevalence; it was obtained by dividing, in the MedCalc online software, the number of children positive either for KK or POC-CCA in each school by the total number of children of that school; 95% CI^b^: 95% confidence interval associated to the mean of infection intensity; it was obtained by dividing, in the MedCalc online software, the sum of EPG or the quantity of CCA in children positive either for KK or POC-CCA in each school by the total number of children who were positive to the corresponding test in that school; df: degree of freedom.

Seventy nine (8.3%) boys were positive for KK against 50 (6%) girls (Table 3). No significant difference (χ^*2*^ *= 3*.*4*, *p = 0*.*06*, *df = 1*) was observed between the prevalence in boys and girls (Table 3). The highest number of positive KK (11.2%) was found in children above 10 years and the lowest (6.3%) in those between 5 and 10 years. Between age groups, significant difference (χ^*2*^ *= 10*.*04*, *p = 0*.*002*, *df = 1*) was observed in the prevalence of *S*. *mansoni* (Table 4).

**Table 3:** Prevalence and infection intensity of *S*. *mansoni* infections according to sex.

Sex	NUA	POC-CCA+ (%)	95% CI^a^	Mean CCA value (mv) ± SD	95% CI^b^	NSA	KK+ (%)	95% CI^a^	Mean EPG ± SD	95% CI^b^
**Male**	1188	638(53.7)	49.6–58	177.3±151.1	176.2–178.5	946	79(8.3)	6.6–10.4	404±820.1	399.6–408.5
**Female**	1065	519 (48.7)	44.6–53.1	190.7±154.8	189.6–191.7	827	50 (6)	4.4–7. 9	497.2±1037.5	491.1–503.5
**P-value**		0.018^a^		0.14^c^			0.06^a^		0.57^c^	
**χ** ^**2**^		5.5, df = 1		-			3.4, df = 1		-	
**t**		-		1.4			-		0.5	

NUA: Number of urine samples analysed; NSA: Number of stool samples analysed; POC-CCA+: Number of children positive for POC-CCA test; KK+: KK: Number of children positive for the Kato Katz; CCA: Circulating cathodic antigen; EPG: egg per gram of stool; SD: Standard deviation; mv: millivolts; ^a^: P-value for chi-square test; ^c^: P- value for Student’s *t*-test. 95% CI^a^: 95% confidence interval associated to prevalence; it was obtained by dividing, in the MedCalc online software, the number of girls or boys positive either for KK or POC-CCA by the total number of girls or boys. 95% CI^b^: 95% confidence interval associated to the mean of infection intensity; it was obtained by dividing, in the MedCalc online software, the sum of EPG or the quantity of CCA in girls or boys positive either to KK or POC-CCA by the total number of girls or boys who were positive to the corresponding test; df: degree of freedom.

**Table 4:** Prevalence and infection intensity of *S*. *mansoni* infections according to age groups.

Age group	NUA	POC-CCA+ (%)	95% CI^a^	Mean CCA value (mv) ± SD	95% CI^b^	NSA	KK (%)	95% CI^a^	Mean EPG ± SD	95% CI^b^
**[5–10]** **[11–15]**	1809 444	885(48.9) 272 (61.3)	45.75–52.2 54.2–68.9	175.52 ± 147.9 214.59 ± 166.1	174.6–176.4 212.8–216.3	1426 347	90 (6.3) 39 (11.2)	5.08–7.7 7.99–15.3	424.8 ± 906 475.69 ± 922.7	420.55–429.1 468.87–482.6
**P-value**		< 0.0001^a^		0.004^c^			0.002^a^		0.66^c^	
**χ** ^**2**^		21.72		-			10.04		-	
**t**		-		3.69			-		0.29	

NUA: Number of urine samples analysed; NSA: Number of stool samples analysed; POC-CCA+: Number of children positive for the POC-CCA test; KK+: Number of children positive for the Kato Katz; CCA: Circulating cathodic antigen; EPG: egg per gram of stool; SD: Standard deviation; mv: millivolts; ^a^: P value for chi-square test; ^c^: P value for Student’s *t*-test. 95% CI^a^: 95% confidence interval associated to prevalence; it was obtained by dividing in the MedCalc online software, the number of children positive either for KK or POC-CCA in each age group by the total number of children in that age group. 95% CI^b^: 95% confidence interval associated to the mean of infection intensity; it was obtained by dividing, in the MedCalc online software, the sum of EPG or the amount of CCA in children positive either for KK or POC-CCA in each age group by the total number of children who were positive to the corresponding test in that age group, df: degree of freedom.

#### Infection intensity of Schistosoma mansoni inferred from Kato Katz

Of the 129 children with *S*. *mansoni* eggs, 64 (49.6%), 32 (24.8%) and 33 (25.6%) were found respectively with light, moderate and heavy infection intensity. The highest infection intensity of 617.8 ± 1150.9 egg per gram of faeces (EPG) was recorded in the public school of Carrière and the lowest in the public school of Makenene group 2 (Table 2). No significant difference (*F = 1*.*5*, *p = 0*.19, df *= 5)* was observed in the infection intensity between schools (Table 2).

In boys, the mean infection intensity was 404 ± 820.1 EPG (95% CI 399.6–408.5) against 497.2 ± 1037.5 EPG (95% CI 491.1–503.5) in girls (Table 3). However, no significant difference (*F = 0*.*5*, *p = 0*.*57*, *df = 1*) was observed in the infection intensities between boys and girls (Table 3).

The mean of infection intensities were *475*.*69 ± 922*.*7* EPG and 134.4 ± 194.6 EPG respectively for school-aged children above 10 years and those of 5 to 10 years. Comparing the infection intensities, no significant difference (*t = 0*.*29*, *p = 0*.*66*, *df = 1)* was also observed between age groups (Table 4).

### Prevalence and infection intensity of *S*. *mansoni* inferred from POC-CCA test

#### Prevalence of S. mansoni infection inferred from POC-CCA test

Of the 2253 urine samples that were analysed with POC-CCA test and for which the results were generated either by the reader or the visual scoring system, 1157 were positive; giving thus a prevalence of 51.3%. The highest prevalence of 72.6% was recorded in the public school of Carrière (95% CI: 64.2–81.7) and the lowest prevalence of 34.4% (95% CI: 23.5–48.5) in the public school of Kinding Nde (Table 2). Comparing the prevalence of *S*. *mansoni* infections revealed by POC-CCA, significant difference (*χ*^*2*^ *= 137*.*1*, *p < 0*.*0001*, *df = 9*) was observed between schools (Table 2). The values of the prevalence were significantly (χ^*2*^ *=* 5.57, *p = 0*.*018*, *df = 1*) different between boys (53.7%) and girls (48.7%) (Table 3), and also between age groups (χ^*2*^ *= 21*.*72*, *p <* 0.0001, *df = 1*) (Table 4). The percentage of positive POC-CCA was 61.3% in children above 10 years and 48.9% in those of 5 to 10 years (Table 4).

#### Infection intensity of S. mansoni inferred from POC-CCA test

From 1157 children who were positive to POC-CCA, visual scoring revealed 512 (44.2%), 162 (14%), 259 (22.4%) and 224 (19.4%) with infection intensities classified respectively as "trace“, 1+, 2+ and 3+. With the reader, the infection intensities varied from 40.02 mv to 720.27 mv. The highest value of infection intensity of 257.4 ± 154.5 mv (95% CI: 255.5–259.3) based on the reader was obtained in children of the public school of Carrière and the lowest value of infection intensity of 80.4±46.5 mv (95% CI: 78.0–82.8) in those of the public school of Kinding Ndjabi (Table 2). Comparing the values of infection intensities, significant difference (*F =* 28.3, *p < 0*.*0001*,df = 9) was observed between schools (Table 2). However, no significant difference (*t = 1*.*48*, *p = 0*.*14*, *df = 1*) was observed between boys (177.3 ± 151.1 mv) and girls (190.7 ± 154.8 mv) (Table 3).

The infection intensities of *214*.*59 ± 166*.*1* mv and 175.52 ± 147.9 mv were obtained respectively in school-aged children of above 10 years and those of 5 to 10 years. Comparing values of infection intensities, significant difference (*t = 3*.*69*, *p = 0*.*004*, *df = 1*) was observed between age groups (Table 4).

### Comparison between results of Kato-Katz and those of POC-CCA

From the 1709 urine and stool samples both analyzed for the presence of *S*. *mansoni* eggs and CCA, concordant results between KK and POC-CCA were observed for 953 (55.76%) samples for which 128 (7.49%) and 825 (48.27%) were respectively positive and negative for both tests. Discordant results were obtained for 756 (44.24%) samples that were all positive for POC-CCA, but negative for the KK test. All children positive for the KK test were also positive for POC-CCA. The concordance index expressed as the Kappa coefficient was 0.14 (*P < 0*.*0001*; 95% CI: 0.12 to 0.16); indicating a fair strength of agreement between results of KK and those of POC-CCA. The infection intensities resulting from KK were comparable to those of POC-CCA. For instance, out of the 33 children having heavy infection intensities inferred from KK, 29 had high CCA levels and were visually scored as 3+ based on POC-CCA test. Comparing results of infection intensities inferred from these two tests, a significant positive correlation (rho Spearman’s correlation coefficient = 0.38; p < 0.0001) was obtained between the infection intensities of *S*. *mansoni* recorded by KK and POC-CCA.

The sensitivity, specificity and accuracy of POC-CCA in comparison to KK were 100% (95% CI 83.4–118.9), 52.2% (95% CI 48.7–57.9) and 55.8% (95% CI 52.3–59.4), respectively.

### Spatial distribution of *S*. *mansoni* infection and its infection intensity

**Fig 2:**
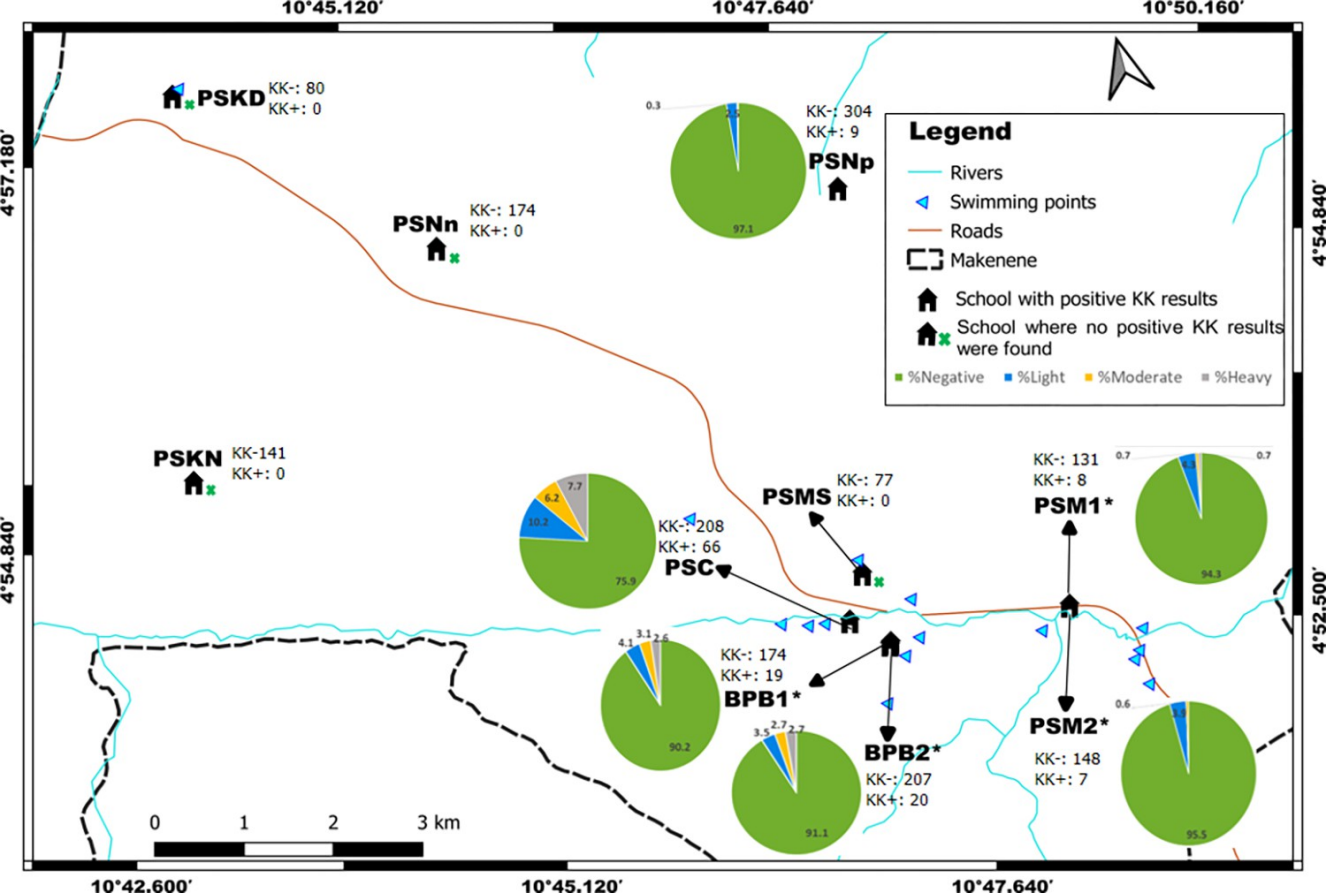
Distribution per school of the percentage of children with no infection or carrying different infection intensities inferred from Kato-Katz (base layer of the maps were obtained using a free online spatial data software (https://www.diva-gis.org/gdata). BPB1: Government bilingual primary school of Baloua group 1; BPB2: Government bilingual primary school of Baloua group 2; PSM1: Public school of Makenene group 1; PSM2: Public school of Makenene group 2; PSNn: Public school of Nyokon; PSKD: Public school of Kinding Nde; PSKN: Public school of Kinding Ndjabi; PSMS: Public school of Mock-Sud; PSC: Public school of Carrière; PSNp: Public school of Ngokop, *: Schools in the same building. KK+: Number of children carrying *S*. *mansoni* eggs in each school; KK-: Number of children without *S*. *mansoni* egg in each school.

**Fig 3:**
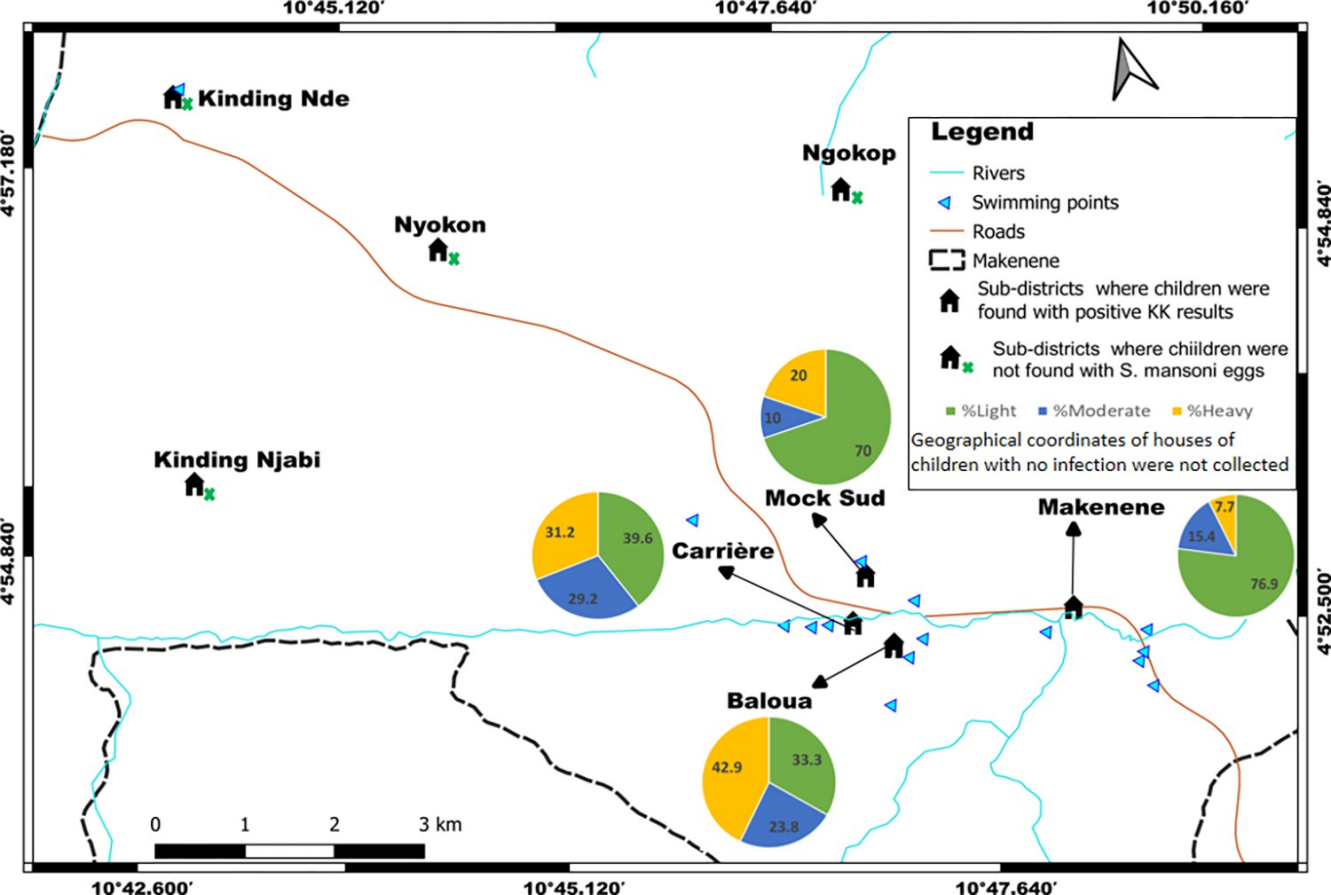
Sub-districts distribution of the percentage of children carrying different infection intensities inferred from Kato-Katz (base layer of the maps were obtained using a free online spatial data software (https://www.diva-gis.org/gdata).

Out of 1157 children who were positive to POC-CCA, houses’ geographical coordinates were recorded for 737 of them; 332, 104, 160 and 141 of these children had their POC-CCA cassettes scored as “Trace”, 1+, 2+ and 3+, respectively. These coordinates included those of 92 children who were found with eggs in their stools. Out of the 92 children carrying *S*. *mansoni* eggs in their stool, 43, 22 and 27 had respectively light, moderate and heavy infection intensities. In the 737 houses recorded, 100 of them had two or more than two infected children. Houses of children having eggs in their stools are concentrated around four schools: Public school of Carrière (PSC), government bilingual primary school of Baloua groups 1 and 2 (BPB1 and BPB2) and public school of Mock-Sud (PSMS) (Fig 2). These schools are located in three sub-districts named Carrière, Baloua and Mock Sud. Moreover, houses of children having heavy infection intensities were recorded in sub-districts Baloua, Carrière and Mock-sud where risky biotopes such as swimming points were identified (Fig 3). In the contrary, the houses of children having light and moderate infection intensities were recorded in all sub-districts (Fig 3). No child with *S*. *mansoni* egg was from the public school of Kinding Nde (PSKD), the public school of Kinding Ndjabi (PSKN), the public school of Nyokon (PSNn) and the public school of Ngokop (PSNp). These schools are located in sub-districts named Kinding Nde, Kinding Ndjabi, Nyokon and Ngokop.

**Fig 4:**
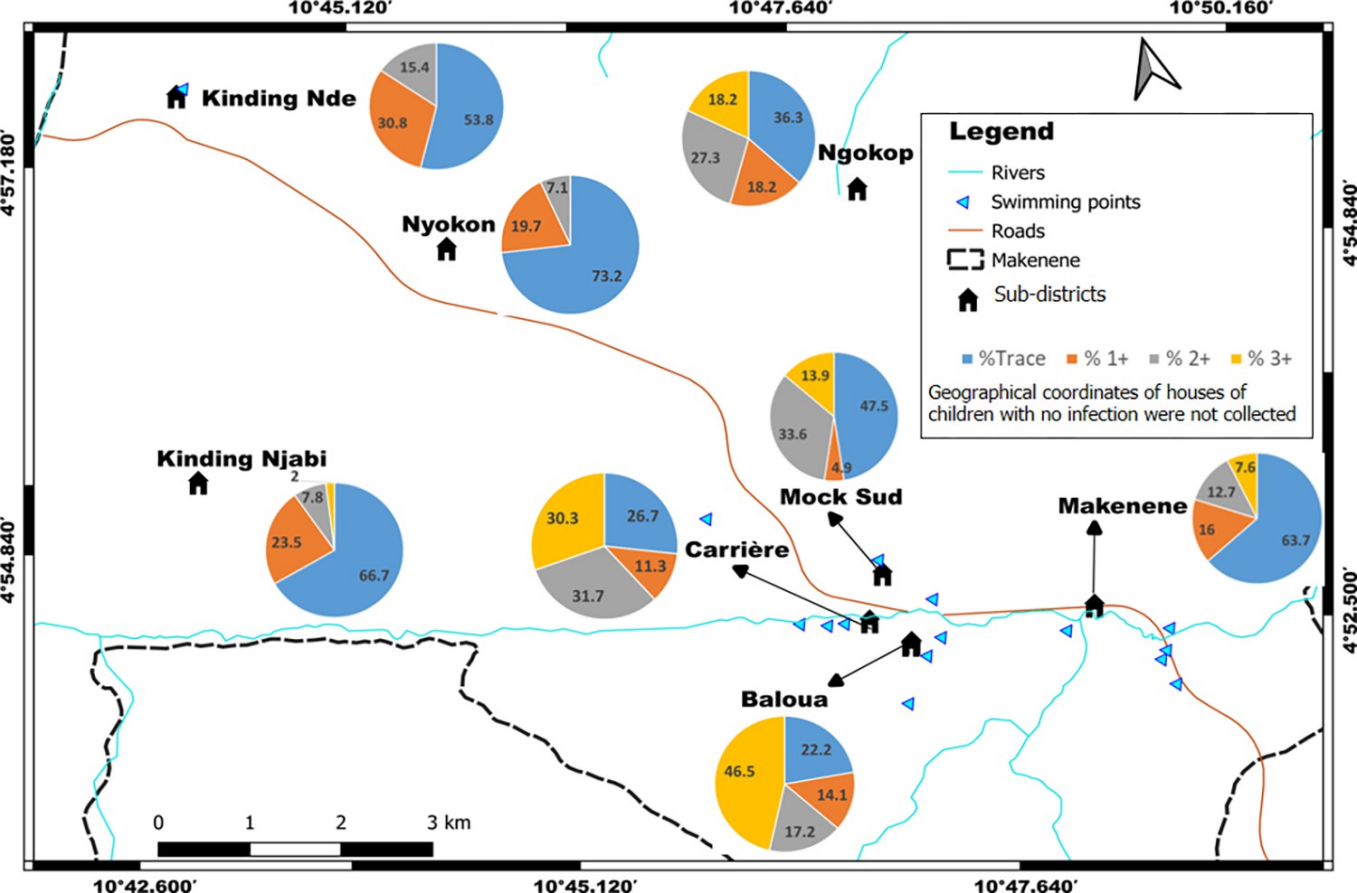
Sub-districts distribution of the percentage of children carrying different infection intensities inferred from POC-CCA (base layer of the maps were obtained using a free online spatial data software (https://www.diva-gis.org/gdata).

Houses of children who were positive to POC-CCA were recorded in all the 8 sub-districts (Fig 4). Those of children having positive KK were recorded only in 4 sub-districts (Fig 3).

**Fig 5:**
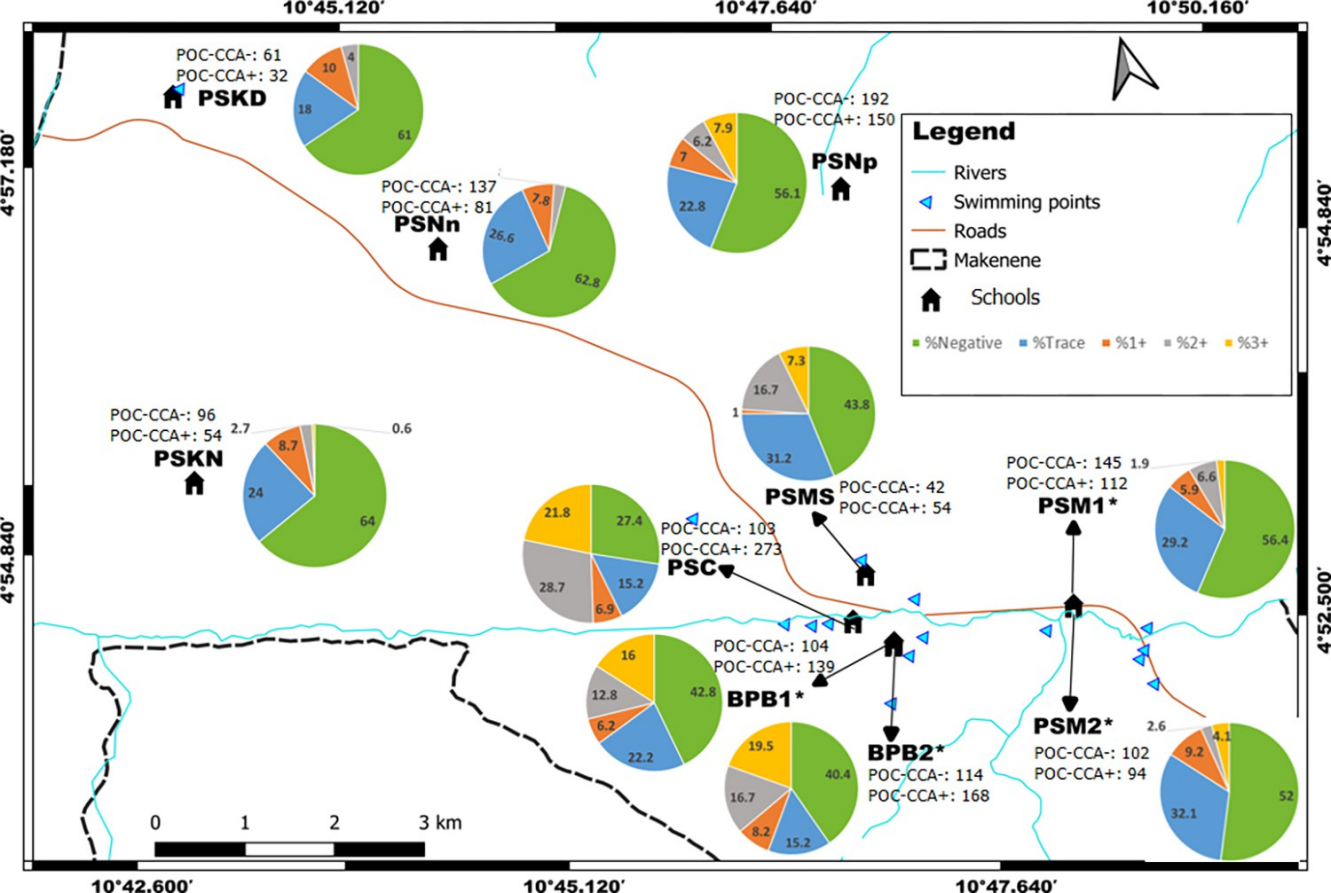
Distribution per school of the percentage of children with no infection or carrying different infection intensities inferred from POC-CCA (base layer of the maps were obtained using a free online spatial data software (https://www.diva-gis.org/gdata). BPB1: Government bilingual primary school of Baloua group 1; BPB2: Government bilingual primary school of Baloua group 2; PSM1: Public school of Makenene group 1; PSM2: Public school of Makenene group 2; PSNn: Public school of Nyokon; PSKD: Public school of Kinding Nde; PSKN: Public school of Kinding Ndjabi; PSMS: Public school of Mock-Sud; PSC: Public school of Carrière; PSNp: Public school of Ngokop, *: Schools in the same building. POC-CCA+: Number of children positive to POC-CCA in each school; POC-CCA-: Number of children with negative POC-CCA in each school.

**Fig 6:**
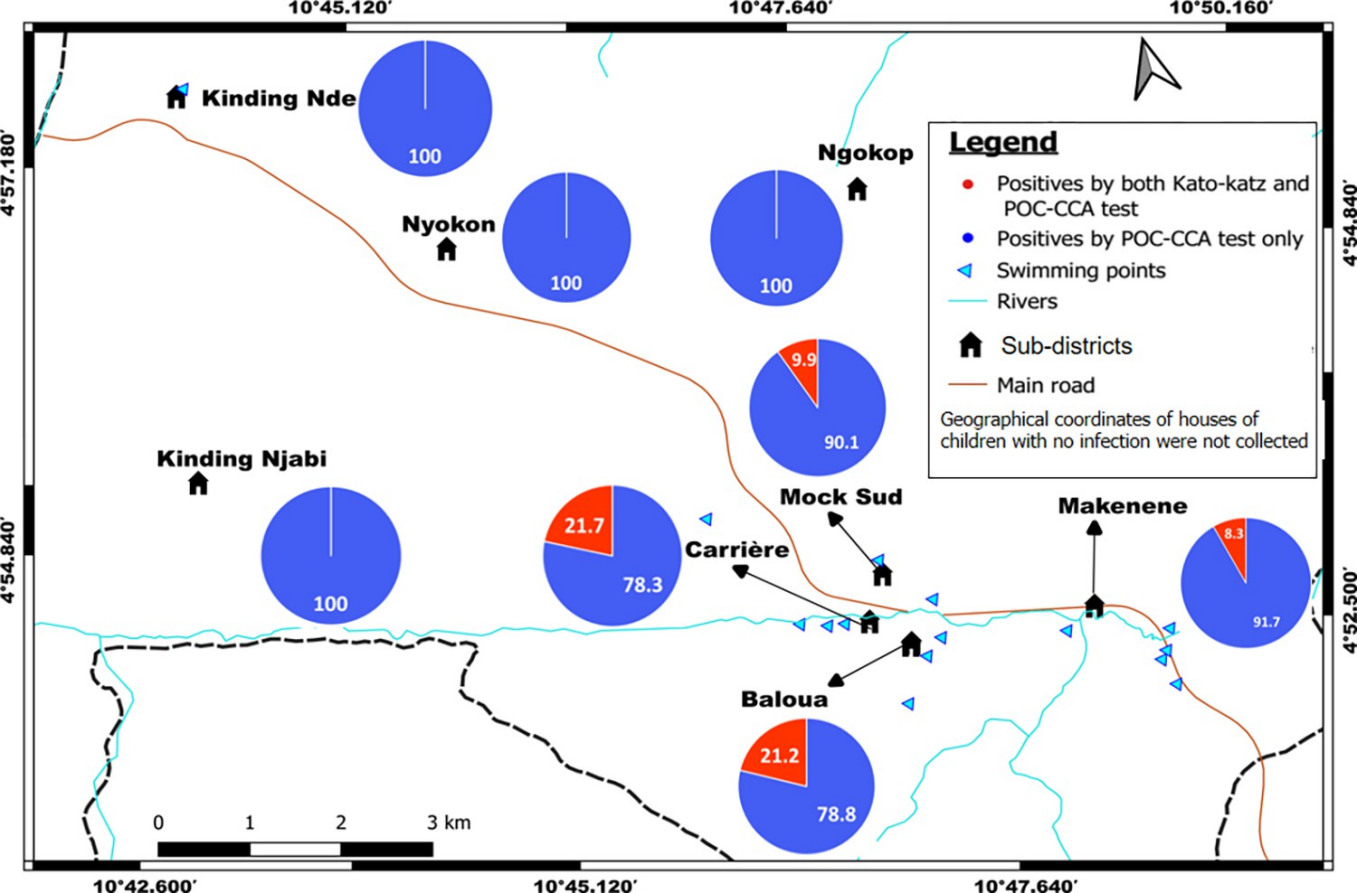
Sub-districts distribution of the percentage of children with *S*. *mansoni* infections based on POC-CCA and KK results (base layer of the maps were obtained using a free online spatial data software (https://www.diva-gis.org/gdata).

Children having heavy infection intensities (3+ scores) lived in four sub-districts (Baloua, Carrière, Mock-Sud and Ngokop) characterized by the presence of swimming sites (Fig 4). However, those having light infection intensities (scored as “trace” and 1+) were identified in all sub-districts, even in sub-districts where no child was found with egg in its stool. When the levels of infection intensities were compared, children carrying heavy infections intensities (scored as +3) were predominant in five schools: public school of Carrière (PSC), government bilingual primary school of Baloua groups 1 and 2 (BPB1 and BPB2), public school of Mock-Sud (PSMS) and public school of Ngokop (Fig 5). These schools were located in sub-districts Carrière, Baloua, Mock Sud and Ngokop. When results from KK and POC-CCA were mapped together, houses of children positive for both tests were from sub-districts Carrière, Baloua, Makenene and Mock Sud (Fig 6). However, children who were positive only by POC-CCA were recorded in all sub-districts (Fig 6). Although children carrying eggs of *S*. *mansoni* lived in four sub-districts, the distribution of infection intensities revealed by KK varied according to these sub-districts (Fig 3). These variations were also observed in schools attended by children carrying eggs of *S*. *mansoni* (Fig 2).

### Clustering of houses of infected children according to infection intensities

**Fig 7:**
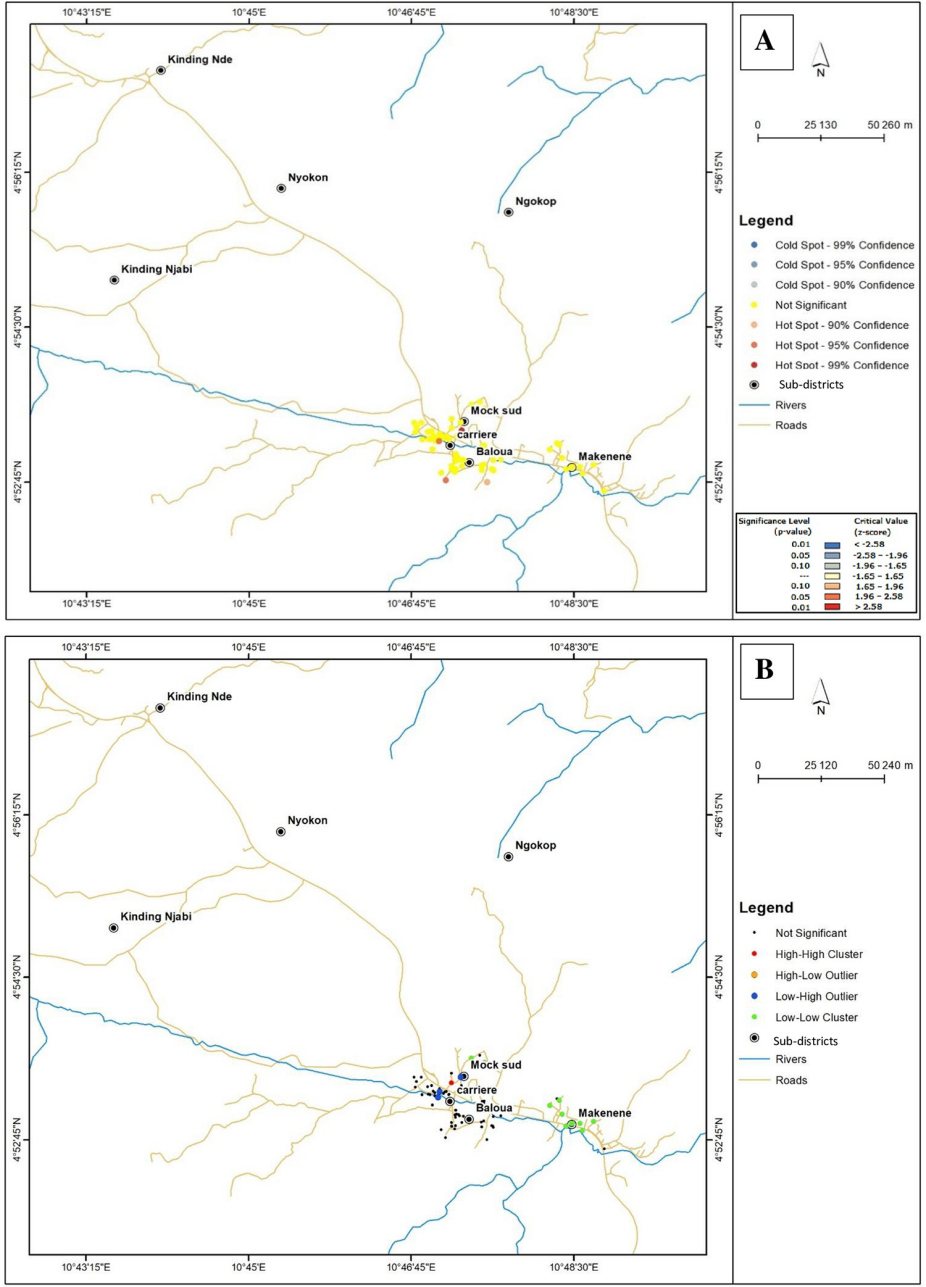
Global and Local Moran’s I cluster maps showing *S*. *mansoni* infection intensities inferred from KK (base layer of the maps were obtained using a free online spatial data software (https://www.diva-gis.org/gdata). High-high cluster indicates significant (*p <* 0.05) clustering (hot-spot) of houses of children carrying heavy infection intensities of *S*. *mansoni*; low-low cluster indicates significant (*p <* 0.05) clustering (cold spot) of houses of children carrying light infection intensities of *S*. *mansoni*; low-high outlier indicates areas where houses of children carrying heavy infection intensities were surrounded by those of children carrying light or moderate infection intensities; high-low outlier indicates areas where houses of children carrying light infection intensities were surrounded by those of children carrying heavy infection intensity.

**Fig 8:**
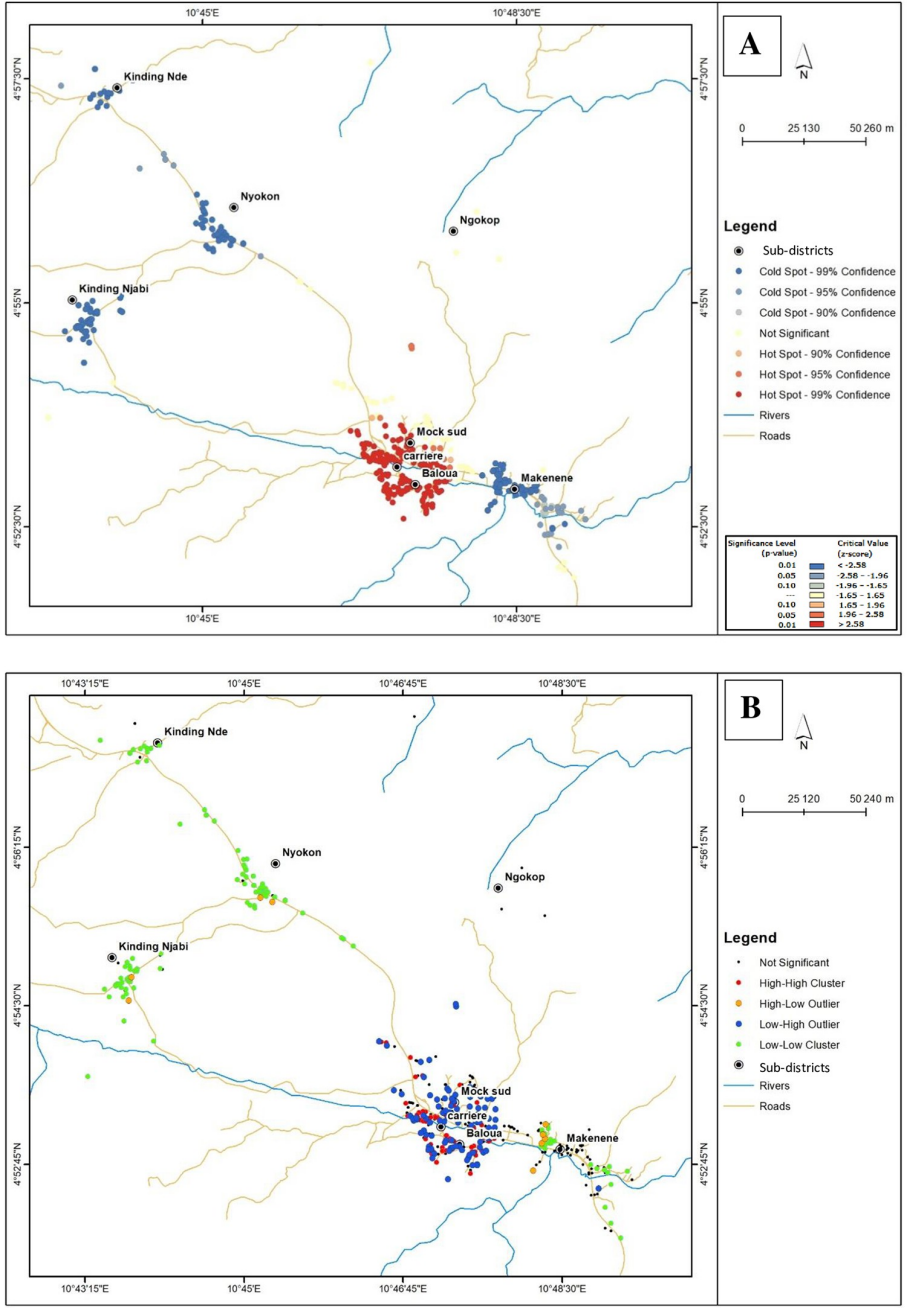
Local Moran’s I cluster maps showing *S*. *mansoni* infection intensities inferred from POC-CCA (base layer of the maps were obtained using a free online spatial data software (https://www.diva-gis.org/gdata). High-high cluster indicates significant (*p <* 0.05) clustering (hot-spot) of houses of children carrying heavy infection intensities of *S*. *mansoni*; low-low cluster indicates significant (*p <* 0.05) clustering (cold spot) of houses of children carrying light infection intensities of *S*. *mansoni*; low-high outlier indicates areas where houses of children carrying heavy infection intensities were surrounded by those of children carrying light or moderate infection intensities; high-low outlier indicates areas where houses of children carrying light infection intensities were surrounded by those of children carrying heavy infection intensity.

The Moran I correlation coefficient of 0.02 with a *P value* (0.66) not significant for KK data indicates an absence of global clustering of houses of children carrying *S*. *mansoni* eggs (Fig 7A). Map of global clustering generated from KK results reveals no hot or cold spots (no significant clustering; *P > 0*.*05*) for *S*. *mansoni* transmission (Fig 7A). However, the significant values of Moran I correlation coefficient (I = 0.36; *P* < 0.0001) resulting from POC-CCA data indicate an overall clustering of houses of infected children (Fig 8A). Map of global clustering generated from POC-CCA results shows hot spot (significant clustering; 95% to 99% confidence; *P < 0*.*05*) for *S*. *mansoni* transmission in Baloua, Carrière and Mock sud sub-districts (Fig 8A). This map also shows cold spots (significant clustering; 95% to 99% confidence; *P < 0*.*05*) for *S*. *mansoni* transmission in Kinding Nde, Kinding Ndjabi, Nyokon and Makenene sub-districts (Fig 8A).

Map of local spatial autocorrelation generated from POC-CCA and KK results revealed four different categories of clusters: high-high, low-low, high-low and low-high (Figs 7B and 8B). The high-high cluster indicates significant clustering (*P <* 0.05) of houses of children carrying heavy infection intensities while the low-low cluster indicates significant clustering (*P <* 0.05) of houses of children carrying light infection intensities. The low-high cluster indicates areas where houses of children carrying heavy infection intensities were surrounded by those of children carrying light or moderate infection intensities. The high-low cluster indicates areas where houses of children carrying light infection intensities were surrounded by those of children carrying heavy infection intensities.

From the KK results, none of the clusters indicated above was recorded in different sub-districts except Makenene where low-low cluster was found (Fig 7B).

Regarding POC-CCA results, high-high clusters (hot-spot transmission sites) were recorded in Carrière, Mock sud and Baloua sub-districts while low-low clusters (cold spot transmission sites) were recorded in Kinding Nde, Kinding Ndjabi, Nyokon and Makenene sub-districts (Fig 8B). Low-high clusters were also found in Carrière, Mock sud and Baloua sub-districts. High-low clusters were found mainly at Makenene sub-district (Fig 8B). No cluster was recorded in the Ngokop sub-district.

### Relationship between the mean distances of houses of infected children and the nearest water points

The mean distances between the nearest water point and houses of infected children vary according to infection intensities revealed by POC-CCA (Table 5). The lowest mean distance of 0.59 ± 2.03 km was found for houses of children carrying heavy (scored as 3+) infection intensities. The highest mean distance of 1.54 ± 2.63 km was obtained for houses of infected children carrying light (scored as 1+) infection intensities (Tables 5 and S1). Comparing the mean distances of houses of infected children according to infection intensities, significant difference (F = 9.50, p < 0.0001) was observed. Children carrying heavy infection intensities lived very close to water point.

**Table 5:** Variations between the mean distances of houses of infected children to the nearest water points according to infection intensities.

Infection intensity	NCHSII	Mean CCA (mv)	Mean DHICWP (Km)
*Based on POC-CCA test*			
Trace	332	66.73 ± 27.74	1.46 ± 2.23
1+	104	128.65 ± 49.59	1.54 ± 2.63
2+	160	227.06 ± 71.64	0.70 ± 1.16
3+	141	442.24 ± 102.31	0.59 ± 2.03
P-value			< 0.0001
F			9.50
*Based on Kato Katz test*	**NCHSII**	**Mean EPG**	**Mean DHICWP (Km)**
Light	43	41.86 ± 26.3	0.34 ± 0.17
Moderate	22	190.9 ± 61.29	0.28 ± 0.18
Heavy	27	1385.77 ± 1364.62	0.26 ± 0.15
P-value			0.14
F			2.01

DHICWP: distance between houses of infected children and the nearest water point; NCHSII: number of children harboring the same infection intensities and for which coordinates of their houses have been projected; Mean CCA: Mean value of circulating cathodic antigen; mv: millivolts; Mean EPG: Mean egg per gram of faeces, Km: kilometers.

Regarding KK results, the mean distances of houses of children carrying *S*. *mansoni* eggs to the nearest water point vary from 0.26 ± 0.15 km (for heavy infection intensities) to 0.34 ± 0.17 km (for light infection intensities) (Table 5). However, when these distances were compared, no significant difference (F *= 2*.*01*, *p = 0*.*14*) was observed between the mean distance of houses of children carrying *S*. *mansoni* eggs and the nearest water point.

### Correlation between the mean distances of schools of infected children and the nearest water points

For this correlation, data of schools located on the same site were combined. It was the case for the government bilingual primary school of Baloua group 1 and group 2 and the public school of Makenene group 1 and group 2. The correlation coefficients between the number of children bearing different infection intensities and the distances of schools to the nearest water points vary from -0.16 to 0.009 for POC-CCA and from -0.06 to 0.04 for KK (Table 6). However, no significant correlation was observed between the distance of schools to the nearest water points and the number of children bearing different infection intensities neither for POC-CCA nor for KK (Table 6).

**Table 6:** Correlation coefficients between the mean distances of schools to the nearest water points and the infection intensities of *S*. *mansoni*.

Infection intensities	*P*	*F*	*r* ^2^
*Based on POC-CCA test*			
Trace	0.92	0.008	-0.16
1+	0.92	0.009	-0.16
2+	0.34	1.07	0.009
3+	0.38	0.86	-0.01
*Based on KK*			
Light	0.46	0.62	-0.06
Moderate	0.36	0.97	-0.004
Heavy	0.28	1.36	0.04

*r*^2^: correlation coefficient, F: F-static from linear model test, P: P-value

## Discussion

Providing data on the spatial distribution of schistosome infections is important for the monitoring of control measures that will lead to WHO goal of eliminating schistosomiasis as a public health problem by 2030. The identification of *S*. *mansoni* infections either by KK or POC-CCA confirmed that Makenene remains endemic for schistosomiasis despite about 15 years of mass administration of PZQ to school-aged children. As KK has been used in several studies, the current prevalence of *S*. *mansoni* infection of 7.3% inferred from KK is too low compared to 23.6% and 49% reported 7 and 25 years ago in the same locality [16,18]. Our results are in agreement with the reduction of schistosomiasis prevalence that has been widely reported in most endemic regions. Similar reductions have been reported not only in an endemic area of Cameroon between 1985 to 2010 (reduction of the disease prevalence from 81.60% to 41%) after the implemention of annual mass administration of PZQ to school-aged children in 2007 [18], but also in the Yumbe district in the northwest of Uganda where the prevalence of *S*. *mansoni* infections decreased from 21.1% to 6.3% following two annual rounds of mass administration of PZQ [29]. Regardless what diagnostic test was used, the current schistosomiasis prevalence is low compared to the value of 69.8% reported 8 years ago with POC-CCA in the same locality [30].

The differences observed between results of samples tested positive for POC-CCA, but negative for the KK test could be explained by the ability of POC-CCA to identify individuals carrying young worms not excreting eggs yet [31–34]. The daily fluctuations in the number of eggs excreted and/or the heterogeneous distribution of eggs in stool samples are additional factors that could explain some negative KK tests in samples tested positive for POC-CCA [35,36]. In addition to that, it is likely that some children harboring low infection intensities have probably passed undetected because only one KK slide was prepared and examined from a stool sample collected [31–34,36,37]. Although KK test can miss some infections and especially in individuals harboring low intensity of schistosome infections, it has the advantage to be direct, specific, low-cost, relatively simple to perform under field conditions with limited health facilities and to detect other helminthes infections [11,35,38]. The ability of POC-CCA to detect infections in individuals who were tested negative for KK makes POC-CCA a reliable diagnostic tool for assessing the prevalence of schistosomiasis, especially in low endemicity settings. POC-CCA also has the advantage to be used on non-invasive samples such as urine and to detect infections in which worms have not yet starting laying egg [21,33]. The low Kappa coefficient of 0.14 indicates a fair strength of agreement between results of KK and those of POC-CCA. Moreover, the positive correlation observed between the infection intensities inferred from KK (EPG) and POC-CCA (amount of CCA in millivolts) is in agreement with results obtained in previous studies [21,31,34]. It indicates that quantifying CCA on POC-CCA strips can be used to estimate the infection intensities of *S*. *mansoni*.

The significant differences observed in the prevalence and infection intensities of *S*. *mansoni* between schools could be explained by the localization of some of these schools in sub-districts hosting biotopes having favorable bio-ecological conditions for schistosomiasis transmission such as the presence of river, swimming points and sites for washing and/or fetching water. Moreover, the high prevalence reported in school-aged children attending the public school of Carrière followed by the government bilingual primary school of Baloua groups 1 and 2 could also be explained by the close proximity of these schools to the Mock River that has been reported to host important population of *Biomphalaria pfeifferi*, the intermediate host of *S*. *mansoni* [18]. In such environment, the probability for school-aged children to be in contact with infected snails and to acquire schistosome infections is higher. Nevertheless, when the distances of school to the nearest water point was considered, no correlation was obtained with infection intensities; indicating that the proximity of school to water point seems to have no impact on the ability of children to harbor light, moderate or heavy infection intensities of *S*. *mansoni*. These results indicate that the risk for a child to be infected and to carry different infection intensities of *S*. *mansoni* seems to depend not from the distance between schools and the nearest water point, but probably from other factors such as the proximity of houses of infected children with risky biotopes. Mapping schistosome infections and their infection intensities according to the houses of infected children could provide a clear picture of what happens in different sub-districts of an endemic area.

Our results highlighting significantly high prevalence of *S*. *mansoni* infections in boys compared to girls are in agreement with those of previous studies [30,39–45]. These results could be related to observations made during field surveys where boys were swimming and playing most often in water compared to girls. In such context, boys are more exposed to infected snails and therefore, have an increased risk of acquiring and to be re-infected by schistosomes [46]. Nevertheless, in one of our recent publications, contradictory results were reported in the same endemic area [21]. The discrepancies between results of these studies could be explained by the sample size, the involvement of other sub-districts in which boys’ behavior differs from that of girls. The present study involved ten schools of 8 sub-districts while in that of Mewamba et al. [21], only three closely related schools of three sub-districts were enrolled. Mixing up results of several sub-districts as it was done in the present study has probably provided an overall epidemiological picture of what happens in this endemic area. However, such mixing up has probably led to miss-interpretation of what happens in each sub-district or in closely related sub-districts.

As already reported in previous studies [30,43,45,47–49], the significantly high *S*. *mansoni* prevalence in children above 10 years compared to younger ones (10 years and less) could be explained by the fact that the first group of children (those above 10 years) seems to be more involved in risky activities such as washing clothes, fetching water, swimming and bathing in areas where infected snails could be found [50]. Performing regularly such activities increases the probability for these children to be re-infected and therefore, to be heavily infected.

The spatial distribution of *S*. *mansoni* infections show that houses of infected children were clustering around the public schools of Carrière, the government bilingual primary school of Baloua groups 1 and 2 and the public school of Mock sud. Located in sub-districts Carrière, Baloua and Mock-Sud, these sub-districts can be considered as the most endemic sub-districts where hot-spots schistosomiasis transmission sites could be found. This hypothesis is strengthened by the presence of risky biotopes such as the river Mock, several swimming sites as well as sites for clothes washing and for fetching water. Moreover, the significant Moran correlation coefficients for the sub-districts Baloua, Carrière and Mock Sud indicate local clustering of children carrying heavy infection intensities in these sub-districts. In addition, maps of global and local clustering of infection intensities of *S*. *mansoni* have shown significant hot spot transmission sites in these sub-districts; strengthening therefore the possibility of having hot-spots transmission sites for schistosomiasis in these sub-districts. Sharing biotopes where infected snails could be found, inhabitants and especially school-aged children living in these sub-districts are probably more exposed to the risk of acquiring schistosome infections. Moreover, the fact that these high endemicity sub-districts are crossed by the Mock river is an additional factor that could increase the risk of acquiring schistosome infections because this river has been reported to contain important population of *Biomphalaria pfefferi* which is known as an intermediate host of *S*. *mansoni* [18]. In addition, the presence of risky biotopes such as swimming sites along the river, sites to wash clothes and fetch water for domestic purposes pleads also in favor of the existence of hot-spots transmission of schistosomiasis. This hypothesis is strengthened by results of the present study showing that the infection intensities increase when the distance between houses of infected children to the nearest water point decreases. These results are in accordance with those of Lamberti et al. [51] reporting that the proximity of houses of infected individuals with risky biotopes can be considered as a significant factor for both *S*. *mansoni* infection and its infection intensities. As most of these risky biotopes were very close to houses of infected children, they are most often visited by school-aged children. These children can easily be in contact with infected snails and in consequence, can acquire or be re-infected by *S*. *mansoni*. In such context, the probability of inhabitants and especially school-aged children to become infected and re-infected is high. As already reported by Arnold et al. [52], children living close to water seem to be at higher risk to become infected and to carry heavy infection intensities of *S*. *mansoni* because they have the possibility to visit more easily and frequently biotopes containing infected snails.

In sub-districts where schistosomiasis transmission was high, there is a great need to boost control operations by treating not only school-aged children, but also adults. In addition, two rounds of treatment with PZQ could be implemented in such sub-districts. Snails’ control, water sanitation and hygiene, the dissemination of information, education, and communication and accurate estimation of the disease prevalence using appropriate diagnostic tools like POC-CCA before treatment are additional elements that could be added to PZQ administration. All these activities are of great importance because high endemicity sub-districts recording hot spot transmission sites have been reported with children harboring heavy infection intensities. As people harboring heavy worm burden have been reported to be responsible of about 80% of eggs released in the environment [53], they strongly contribute to schistosomiasis transmission and consequently, play crucial epidemiological role. In addition to the fact that most infected children live in the same environment, those with heavy infection intensities are clustered together in hot spots. This clustering could be explained by the close proximity of children having heavy infection intensity with risky biotopes. Being permanently in such biotopes, the probability for children to be regularly in contact with infected snails increases. In consequence, these children can probably be more re-infected. In sub-districts reporting low disease prevalence, low infection intensities, or having sites of low transmission (cold spots) and moving towards schistosomiasis elimination, the control measures must be different from those used in other epidemiological settings. Although some sub-districts of low transmission (having cold spots for schistosomiasis transmission) like Kinding Ndjabi move towards the elimination because no child was found with schistosome eggs, the low disease prevalence revealed with POC-CCA indicates that the transmission of schistosomiasis is still ongoing in this sub-district. Despite the fact that the control measures implemented in this sub-district enabled to reduce the disease prevalence and the infection intensities, achieving schistosomiasis elimination in this endemic area requires to also apply control measures in sub-districts presenting low prevalence and light infection intensities. In sub-districts like those showing wide spread of infected children with low and moderate infection intensities, risky biotopes are probably scarce and far away from the houses of infected individuals.

The fact that most infected children with low infection intensity were diagnosed most often with POC-CCA indicates a low disease prevalence in sub-districts where these children live. Our results on POC-CCA are in agreement with those of previous authors recommending this test for reliable surveillance and mapping of schistosomiasis in low endemicity settings [30–32,54,55]. The disparities observed in the prevalence and infection intensities of *S*. *mansoni* in different sub-districts could be linked to different bio-ecological factors that prevail in various sub-districts. With such differences, appropriate control measures need to be designed and implemented according to each epidemiological setting. Face to the complexity of schistosomiasis epidemiology in different bio-ecological settings of Makenene, identical control measures cannot be deployed in different sub-districts. There is a need to design control measures that must be specific and appropriate to each bio-ecological setting. Adapting these control measures will reduce the efforts and costs of control operations that should be deployed to reach schistosomiasis elimination. The control measures that will be implemented in sub-districts presenting high disease prevalence and heavy infection intensity or having hot spot transmission sites must be different to those of low endemicity. Data from fine-scale mapping notably the clustering of infected children as well as those carrying heavy infection intensities in hot spot and in the same sub-districts could help policy makers to identify sub-districts presenting potentially higher transmission of schistosome infections and where control measures must be boosted. In such sub-districts for instance, the control measures implemented to fight against COVID-19 notably regular washing of hands must be maintained. As already performed in the sub-district of Kinding Ndjabi, improving the water quality by constructing wells and water pumps will enable to reduce the contacts between inhabitants and risky biotopes. Additional efforts must be performed to sensitize households and policy makers on how to prevent schistosomiasis transmission.

As far as this study was performed in 10 out of 14 schools of 8 sub-districts of Makenene, some school-aged children have not been taken into account. In addition to that, school-aged children not attending schools have not been also taken into account. Furthermore, the fact that only one KK tick smear was prepared from a single stool sample could have led to an underestimation of *S*. *mansoni* infections in school-aged children of different sub-districts. Data on the snails distribution and their infection have also not been considered in this study. All these factors could play important role to deeply understand the transmission and the epidemiology of schistosomiasis. Considering these factors could enable a better understanding of the disease transmission and therefore, the designing of the most appropriate control measures for achieving schistosomiasis elimination as foreseen by WHO.

## Conclusion

This study confirmed the endemicity of intestinal schistosomiasis in several sub-districts of Makenene. Fine-scale mapping of schistosome infections revealed a clustering of infected children in the same environment. It enabled the identification of sub-districts presenting high disease prevalence and high intensity of *S*. *mansoni* infections. These sub-districts can be considered as having potentially high schistosomiasis transmission risk. Inhabitants living in these sub-districts and especially school-aged children are at high risk of contracting *S*. *mansoni* infections. Fine-scale mapping of schistosome infections and infection intensities enabled to identify high transmission sub-districts where control measures must be boosted to reach schistosomiasis elimination.

## Supporting information

S1 Table Environmental data collected in different sub-districts. (XLSX)
